# Expression of miR-487b and miR-410 encoded by 14q32.31 locus is a prognostic marker in neuroblastoma

**DOI:** 10.1038/bjc.2011.388

**Published:** 2011-10-04

**Authors:** C-H Gattolliat, L Thomas, S A Ciafrè, G Meurice, G Le Teuff, B Job, C Richon, V Combaret, P Dessen, D Valteau-Couanet, E May, P Busson, S Douc-Rasy, J Bénard

**Affiliations:** 1 CNRS UMR 8126, University Paris-Sud 11, Institut Gustave Roussy, 114 rue Edouard, Villejuif F-94805, France; 2 Department of Experimental Medicine and Biochemical Sciences, University of Roma ‘Tor Vergata’, Roma I-00133, Italy; 3 Functional Genomic Platform, Institut Gustave Roussy, Villejuif F-94805, France; 4 Department of Statistics and Epidemiology, Institut Gustave Roussy, Villejuif F-94805, France; 5 INSERM U 590, Centre Léon Bérard, Lyon F-69008, France; 6 Paediatric Department, Institut Gustave Roussy, Villejuif F-94805, France; 7 Medical Biology and Pathology Department, Institut Gustave Roussy, Villejuif F-94805, France

**Keywords:** neuroblastoma, non-*MYCN*-amplified, microRNA, 14q32.31 locus, prognosis

## Abstract

**Background:**

Combination of age at diagnosis, stage and *MYCN* amplification stratifies neuroblastoma into low-risk and high-risk. We aimed to establish whether a microRNA (miRNA) signature could be associated with prognosis in both groups.

**Methods:**

Microarray expression profiling of human miRNAs and quantitative reverse-transcriptase PCR of selected miRNAs were performed on a preliminary cohort of 13 patients. Results were validated on an independent cohort of 214 patients. The relationship between miRNA expression and the overall or disease-free survival was analysed on the total cohort of 227 patients using the log-rank test and the multivariable Cox proportional hazard model.

**Results:**

A total of 15 of 17 miRNAs that discriminated high-risk from low-risk neuroblastoma belonged to the imprinted human 14q32.31 miRNA cluster and two, miR-487b and miR-410, were significantly downregulated in the high-risk group. Multivariable analyses showed miR-487b expression as associated with overall survival and disease-free survival in the whole cohort, independently of clinical covariates. Moreover, miR-487b and miR-410 expression was significantly associated with disease-free survival of the non-*MYCN*-amplified favourable neuroblastoma: localised (stage 1, 2 and 3) and stage 4 of infant <18 months.

**Conclusion:**

Expression of miR-487b and miR-410 shows predictive value beyond the classical high-/low-risk stratification and is a biomarker of relapse in favourable neuroblastoma.

Neuroblastoma, the most common solid tumour of childhood, affects very young children (median age: 17 months at diagnosis). Although neuroblastoma accounts for the highest morbidity and mortality among childhood cancers, some rare forms spontaneously regress, a unique phenomenon in human oncology. Moreover, the wide spectrum of clinical behaviours of neuroblastoma constitutes a model of tumour heterogeneity. Stage, age and *MYCN* amplification represent the basic parameters used for risk stratification for management and treatment of the disease (Maris, 2010). Last decade has dealt with the search of tumour-oriented genomic information that refines pronostic classification of patients. Hyperdiploidy and several genomic aberrations, among which gain or loss of whole chromosomes and intra-chromosomal alterations including loss of 1p, 11q, 14q and gain of 17q (Ambros *et al*, 2003; Maris, 2010) have finally led to pan-genomic DNA analyses whose prognostic values are under investigation (Janoueix-Lerosey *et al*, 2009; Ambros *et al*, 2011). According to the basic clinical stratification, paediatric oncologists distinguish two groups of neuroblastoma at diagnosis, the optimal age cutoff being 18 months (Cohn *et al*, 2009) The low-risk group consists of non-*MYCN*-amplified tumours that occur either as localised forms (stage 1, 2 and 3) or as metastatic forms in the children of age <18 months (stage 4 and 4S). The high-risk group comprises all *MYCN*-amplified neuroblastoma regardless of stage and age of the child, and non-*MYCN*-amplified stage 4 neuroblastoma for children of age ⩾18 months. If the low-risk group elicits up to 90% survival (De Bernardi *et al*, 2009; Rubie *et al*, 2011), the high-risk group shows aggressive tumours leading to death very frequently with a survival around 35% at 5 years. However, relapse for low-risk patients constitutes a current concern. With regards to treatment, low-risk patients benefit essentially of surgery and induction chemotherapy to improve response rates while high-risk patients undergo a systematic induction chemotherapy before surgery, high-dose chemotherapy and retinoic acid-based maintenance treatments (Matthay *et al*, 1999; Valteau-Couanet *et al*, 2005). Thus, in terms of prognosis, current clinical issues are to predict survival for the high-risk group and disease-free survival for the low-risk group in order to optimise therapy. It is commonly admitted that a better knowledge of molecular and genetic mechanisms could be helpful in these respects.

The use of array technologies to characterise microRNAs (miRNAs) has enabled discovering unique miRNA expression patterns related to clinical features of several cancers. The miRNAs are endogenous small non-coding RNAs that regulate gene expression by promoting mRNA degradation or preventing translation by imperfect sequence-specific interaction with the 3′-untranslated region of mRNAs. Deregulation of miRNA expression at a number of loci has already been reported in neuroblastoma. Expression profiling analysis of 157 miRNA's loci in neuroblastoma led to the identification of 32 loci that were differentially expressed in favourable and unfavourable tumour subtypes indicating a potential role of miRNAs in neuroblastoma pathogenesis (Chen and Stallings, 2007). Many of these loci are significantly underexpressed in tumours with *MYCN* amplification (Schulte *et al*, 2008; Bray *et al*, 2009). By deep sequencing analysis of five favourable and five unfavourable *MYCN*-amplified neuroblastoma, Schulte *et al* (2010) reported the underexpression of 20 miRNAs. Lin *et al* (2010) found a global downregulation of miRNA expression in advanced neuroblastoma, and identified 27 miRNAs that clearly distinguish low- from high-risk patients.

To our knowledge, no data are available on the possibility to use deregulated specific miRNA as predictors of relapse for low-risk patients and survival for high-risk patients. In the present work, we show that the downregulation of miR-487b and miR-410, two miRNAs located at the 14q32.31 locus, is associated with the outcome of neuroblastoma in terms of both overall survival and relapse whether *MYCN* is amplified or not. Importantly, our results point out that underexpression of these two miRNAs could predict relapse among neuroblastoma currently classified as low-risk.

## Materials and methods

### Tumour collection

Tumour samples from a retrospective cohort of 227 patients staged according to the International Neuroblastoma Staging System (Brodeur, 2003) were gathered between 1987 and 2009 at the Institut Gustave Roussy (*n*=215) and at the Centre Leon Bérard (*n*=12) with the approval of the appropriate ethic committees and according to the national law on the protection of people taking part in biomedical research. Primary tumour tissues, obtained from patients either by true-cut or after surgery, were immediately snap frozen before being stored in liquid nitrogen until nucleic acids extraction.

### Tumour cryosections and total RNA extraction

The first and last cryosections were used to select tumour tissues with a malignant tumour cell content of ⩾60%. (Ambros *et al*, 2003; Benard *et al*, 2008). Total RNA was isolated using TRIzol Reagent (Invitrogen, Carlsbad, CA, USA) according to the manufacturer's protocols. Nucleic acid concentration and purity were determined by using Nanodrop ND-1000 (Nanodrop, Wilmington, DE, USA), while quality was verified with a 2100 Bioanalyser (Agilent Technologies, Santa Clara, CA, USA).

### miRNA microarray expression analysis

Each sample was prepared according to the Agilent*’*s miRNA Microarray System protocol. Total RNA (100 ng) was labelled and hybridised to Agilent human miRNA 8 × 16-k microarrays (v3) containing 851 human and 88 human viral miRNAs each replicated 16 times, according to the manufacturer's instructions. Agilent miRNA assays integrate eight individual microarrays on a single glass slide. Each microarray includes ∼15-k features containing probes sourced from the miRBASE public database, release 13.0 (Griffiths-Jones *et al*, 2008). The probes were 40–60-mer oligonucleotides directly synthesised on the array with a special design to cope with the small size of miRNA. Indeed, each probe was composed of three features: hybridisation sequence (10–20 nucleotides), a G residue at 5′ end of the hybridisation sequence that complement the 3′ end C residue introduced in labelling and an extended 5′ hairpin abutting on the probe–target region to increase target size and miRNA specificity. The additional G–C pair in the probe–target interaction region stabilises targeted miRNA relative to homologous RNAs. The array also contained a set of repeated positive and negative controls. All processing methods used for miRNA analyses were performed on the Cy3 Median Signal from Agilent Feature Extraction raw data files in R, using functions and package collected in the Bioconductor project (Gentleman *et al*, 2004) as well as customed written routines. Flagged spots as well as control spots were systematically removed, and data were log2 transformed. A quantile normalisation was then performed using the *normalizeBetweenArray* function from R package ‘LIMMA’ (Smyth *et al*, 2005) from the Bioconductor project. The median of each probe for a given miRNA was computed and the corresponding value was assigned to the miRNA. Data were then filtered according to the maximum number of missing values allowed for each miRNA (30%).

Unsupervised hierarchical clusters were computed using the ‘dist’ function from R, using the ‘Euclidian’ method as distance measure. The hierarchical clustering was performed with the ‘hclust’ function from R using the distance matrix previously computed and the ward method.

To assess differentially expressed miRNAs, we first estimated the fold changes and standard errors between two groups of samples by fitting a linear model for each miRNA with the ‘lmFit’ function of LIMMA package (Smyth *et al*, 2005). Then, we applied an empirical Bayes smoothing to the standard errors to the linear model previously computed with ‘eBayes’ function of LIMMA. To extract a table of the top-ranked genes from the linear model fit, we used top Table function from LIMMA. The results were saved in a table file format.

### miRNA real-time reverse-transcription PCR

Total RNA (10 ng) was reverse transcribed on the PTC-100 (MJ Research Inc., Waltham, MA, USA) using the stem-loop reverse transcription (TaqMan MicroRNA Reverse Transcription Kit, Applied Biosystems, Foster City, CA, USA) for three selected miRNAs from the miRNA microarray analysis, hsa-miR-409-3p, hsa-miR-410 and hsa-miR-487b, *vs* RNU-44 (small nuclear RNA) as a control. Real-time PCR amplifications run in duplicate using 2 × FastStart Universal Probe Master (Roche, Mannheim, Germany) were performed on the StepOnePlus RealTime PCR System (Applied Biosystems) according to the MIQE guidelines (Bustin *et al*, 2009). Samples that lacked either a template or reverse transcriptase were used as controls. The relative expression of each miRNA was calculated according to the comparative 2^−ΔΔC_*t*_^ quantification method where ΔC_*t*_=C_*t*_(sample)−C_*t*_(normaliser) and ΔΔC_*t*_=ΔC_*t*_(sample)−ΔC_*t*_(calibrator) (Livak and Schmittgen, 2001). RNU 44 was used as a normaliser and IGR-N-835 cell line (Bettan-Renaud *et al*, 1989) as the miRNA expression level reference, sample and reference being amplified in separate wells.

### Statistical analysis

The quantitative reverse-transcriptase PCR (qRT-PCR) data were analysed using GraphPad Prism software to generate scatter plots, and two-tailed Student *t*-test was used to compare the different neuroblastoma groups. The association between various clinical characteristics and miRNA expression levels were examined by the two-tailed Fisher's exact test.

To determine if miRNA expressions were related significantly to overall and disease-free survival of patients (endpoint of cancer-specific death and relapse, respectively), we performed several analyses. Kaplan–Meier survival curves and univariate and multivariable Cox proportional hazards analyses were performed with SAS software. To generate survival curves, we converted continuous miRNA expression levels measured by qRT-PCR to a dichotomous variable, using as cutoff the expression level of IGR-N-835 cell line taken as one. This cutoff was found to be very close to those computed for the expression of miRNAs that best discriminate patients for overall survival (the time from the diagnosis to the date of death of disease) and for disease-free survival (the time from diagnosis to the date of first appearance of relapse or death). This procedure enabled division of samples into two levels with high and low expression of miRNA (over and under the cutoff point, respectively). We compared survival curves by log-rank test and judged *P*<0.05 significant. We undertook univariate Cox proportional hazards regression in SAS software and judged a significance if *P*<0.05. We examined the joint effect of covariates with Cox proportional hazards regression to ascertain whether miRNAs were independent prognostic factors. Age was dichotomised into groups, less and over 18 months. Stage was dichotomised on the whole neuroblastoma cohort on the basis of metastatic progression, stage 1, 2, 3 *vs* stage 4. Stage 4S was included in stage 1, 2, 3 groups because the prognosis of these patients is usually considered as good.

For all miRNAs, patients were categorised into groups with high and low expression, as described above. All variables with *P*<0.05 in univariate analyses were selected in the multivariable model. To evaluate the predictive ability of the markers, sensitivity and specificity were estimated within 2, 5 and 8 years because the event of interest is time-dependent (Heagerty *et al*, 2000). Sensitivity was defined as the probability that a patient having low expression of 14q32.31 miRNA (below the cutoff) to be in the high-risk group and specificity as the probability that a patient with high expression of 14q32.31 miRNA (above the cutoff) to be in the low-risk group.

## Results

### miRNA expression profiling from patients with opposite outcome

**Figure 1:**
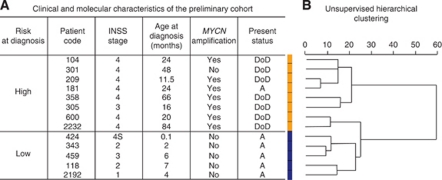
Patients characteristics and tumour clustering using miRNA expression profiling. (**A**) Clinical main characteristics of patients from the preliminary cohort. INSS: International Neuroblastoma Staging System. Present status February 2011: A, Alive; DoD, dead of disease. (**B**) Unsupervised hierarchical clustering. The unsupervised cluster was built by computing a distance matrix (Euclidian distance) between all arrays using the dist() function of R. The distance matrix was then clustered according to ‘ward’ method available in hclust() function of R. The high-risk neuroblastomas are indicated in orange and the low-risk neuroblastomas in blue.

**Figure 2:**
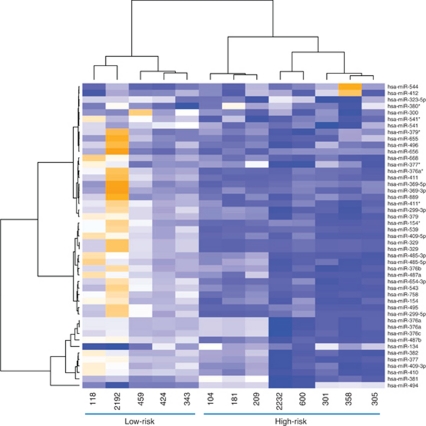
Summary heat map showing the clustering based on 14q32.31 miRNA cluster. Data are represented as two dendrograms. The vertical dendrogram indicates patient sample with closest miRNA expression patterns. The horizontal dendrogram indicates miRNA with closest expression pattern across all patient samples. The Euclidian distance has been used as distance metric, and the clustering is using the ‘ward’ method to gather miRNAs or patient samples together. The heat map colours range from orange (high intensity values) to blue (low intensity values). The miRNAs used for the clustering strongly discriminate the high-risk from the low-risk neuroblastoma. See also Table 1 and Supplementary Table 3.

The search for a miRNA expression signature associated with patient outcome was first performed on a preliminary cohort of 13 primary neuroblastoma tumours whose risk at diagnosis was defined according to clinical criteria (age at diagnosis and stage) and *MYCN* amplification (Figure 1A). This consisted of neuroblastoma with opposite outcomes, namely five low-risk obtained from infants (median age: 3.8 months, median follow-up: 112.6 months) and eight high-risk from children (median age: 27.5 months, median follow-up: 14.8 months). Consistent with this risk stratification, comparative genomic hybridisation array (CGHa) profiles showed chromosomal alterations that were numerical in low-risk and segmental in high-risk neuroblastoma (Supplementary Table 1). As shown in Figure 1B, unsupervised hierarchical clustering on the 851 human and 88 human viral miRNA expression profiling discriminated high-risk from low-risk tumours except for two tumours classified as high risk (patients 600 and 2232). Among the 851 human miRNAs, 50 were differentially expressed in the high-risk relatively to the low-risk group (raw *P*-values <0.05), that is, 37 were downregulated and 13 were upregulated (Supplementary Table 2). In accordance with Chen and Stallings (2007), miRNA-181a and miRNA-181b were found to be upregulated. None of the upregulated miRNAs elicited a significant adjusted *P*-value, whereas 17 downregulated miRNAs displayed highly significant adjusted *P*-values as well as the highest fold change. Strikingly, 15 of the 17 downregulated miRNAs were located at the chromosome 14q32.31 locus (Table 1), a locus that harbours the largest miRNA cluster identified so far in the human genome (Seitz *et al*, 2004). This observation led us to perform an unsupervised hierarchical clustering on the totality of miRNAs encoded at locus 14q32.31. Results efficiently discriminated all high-risk, including patients 600 and 2232, from low-risk (Figure 2 and Supplementary Table 3). As loss of 14q heterozygosity in neuroblastoma was reported (Thompson *et al*, 2001), we performed CGHa that ruled out the possibility that the low expression of 14q32.31 miRNAs in high-risk neuroblastoma was the result of a genomic copy loss (Supplementary Table 1). To confirm microarray data obtained from the preliminary cohort, qRT-PCR was applied to three downregulated 14q32.31 miRNAs, namely miR-487b, miR-410 and miR-409-3p, that were selected because miR-487b and miR-410 showed the highest fold changes and adjusted *P*-values while miR-409-3p ranked in a mean position for both fold change and *P*-value (Table 1). The qRT-PCR analysis confirmed a significant downregulation of these three miRNAs in high-risk compared with low-risk neuroblastoma (Supplementary Table 4). These results suggested a possible association between underexpression of miR-487b, miR-410 and miR-409-3p and risk of death.

### Validation of the low expression of 14q32.31 miRNAs associated with high-risk neuroblastoma

**Figure 3:**
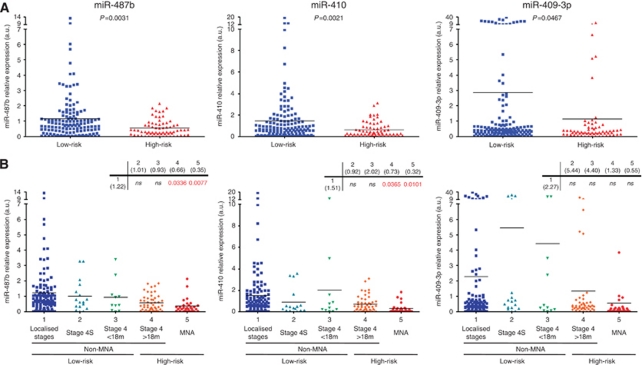
Scatter plots showing the statistical analysis of miR-487b, miR-410 and miR-409-3p expression levels in tissue samples of validation test. (**A**) Validation set tumours (*n*=214) stratified in two groups, low-risk (blue, *n*=142) *vs* high-risk (red, *n*=72). (**B**) Analysis of the whole cohort (*n*=227) stratified according to age, stage and *MYCN*-amplification status (1: *n*=1192: *n*=173: *n*=114: *n*=495:*n*=31). Two-tailed Student *t*-test indicates significance between the two subgroups. Black bars inside the plots indicate the mean value of miRNA expression levels.

To validate the low expression of 14q32.31 miRNAs potentially associated with risk of death, an independent cohort of 214 patients (142 classified as low-risk and 72 as high-risk) was analysed. The main clinical features of patients and tumour characteristics are described in Table 2 and Supplementary Table 4. The expression levels of miR-487b, miR-410 and miR-409-3p were evaluated by qRT-PCR (Supplementary Table 4) relatively to the IGR-N-835 cell line (Bettan-Renaud *et al*, 1989). As presented in the scatter plots, mean expression levels of the three miRNAs were significantly lower in high-risk than in low-risk tumours (Figure 3A). Next, the data were stratified according to age, stage and *MYCN* amplification on the whole cohort of 227 tumours (13 of the preliminary cohort and 214 of the validation set) (Figure 3B). As compared with the non-*MYCN*-amplified localised (stage 1, 2, and 3) tumours, the mean expression levels of miR-487b and miR-410, but not of miR-409-3p, were significantly downregulated in the two high-risk neuroblastoma subgroups, that is, *MYCN*-amplified (*P*<0.01) and non-*MYCN*-amplified stage 4 children aged ⩾18 months (*P*<0.04). These results revealed that the low expression level of miR-487b and miR-410 was significantly associated with the high-risk groups, independently of *MYCN* amplification. Regarding the non-*MYCN*-amplified tumours, it is noteworthy that the average expression values for the three miRNA from stage 4 patients <18 months (*n*=11) were higher than those ⩾18 months (*n*=49) (Figure 3B). The difference was significant for miR-410 and miR-409-3p (*P*=0.0321 and 0.0474, respectively) but not for miR-487b, very likely owing to the small number of stage 4 patients <18 months.

Next, we investigated whether downregulation of the three selected miRNAs was associated with clinical features. To this aim, the statistical analyses were performed on the global retrospective cohort of the 226 clinically documented patients, by converting the continuous miRNA expression values into a dichotomic variable, low and high expression, as described in Materials and methods. Fisher's exact test indicated that, except for miR-409-3p, downregulation of either miR-487b or miR-410 was well associated with stage and *MYCN* amplification (*P*<0.01) (Supplementary Table 5), confirming a link of miR-487b and miR-410, but not miR-409-3p, with the high-risk phenotype. miR-409-3p was thus excluded for further investigations.

### Expression of miR-487b and miR-410 as prognostic marker of patient survival

**Figure 4:**
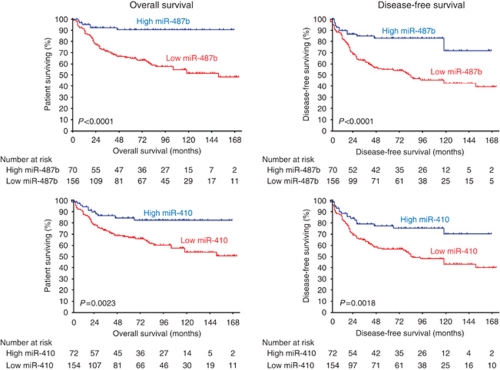
Kaplan–Meier curves for overall and disease-free survival of the whole neuroblastoma cohort (*n*=226) for miR-487b and miR-410 expression. The miRNA expression levels were converted into discrete variables by discriminating the samples into two classes (high and low), under or over the cutoffs.

Statistical analyses were performed to determine the significance of miR-487b and miR-410 expression level as prognostic factor. Disease outcome (survival and relapse events) was known for the 227 patients. The final follow-up date was February 2011 with a median follow-up of 92 months (95% CI=79–104). An event (death or relapse) was registered for 90 patients (40%) with death recorded for 68 patients (30%). An univariate Cox proportional hazards regression showed that patients with low expression of either miR-487b or miR-410 had an overall and disease-free survival significantly lower than patients with high expression (Table 3). Consistent with the univariate Cox regression analysis, Kaplan–Meier survival highlighted that patients with high expression levels of miR-487b and miR-410 displayed a better prognosis than did those with low levels both in terms of overall survival and disease-free survival (*P*<0.0001 and *P*<0.002, respectively, Figure 4). Two multivariable Cox proportional hazard model analyses were performed to assess whether each miRNA was an independent prognostic factor (Table 3). While miR-410 was not statistically associated to overall survival (HR=1.448 95% CI=0.729–2.875, *P*=0.29) and disease-free survival (HR=1.614 95% CI=0.926–2.813, *P*=0.0912), miR-487b was found to be an independent prognostic factor both in overall and in disease-free survival (HR=2.833 95% CI=1.192–6.731, *P*=0.018 and HR=2.145 95% CI=1.164–3.952, *P*=0.014, respectively).

In addition to the strong association between miR-487b with either the overall survival or the disease-free survival measured by the hazard ratios (Table 3), Table 4 showed the sensitivity and the specificity of miR-487b predictive ability. The high sensitivities (⩾0.84) within 2, 5 and 8 years indicated the good ability of miR-487b to discriminate between overall survival and disease-free survival. The specificity giving the probability of patients with high expression of miR-487b to be part of the low-risk group was between 34% and 45%.

### miR-487b and miR-410 expression predict overall and disease-free survival in low-risk neuroblastoma

Next, the association of the two miRNAs with survival was investigated in the group of high-risk patients (*n*=80). Kaplan–Meier curves showed a trend of significance regarding the association of miR-487b or miR-410 expression with overall survival (*P*=0.059) and disease-free survival (*P*=0.067) (Supplementary Figure 1).

**Figure 5:**
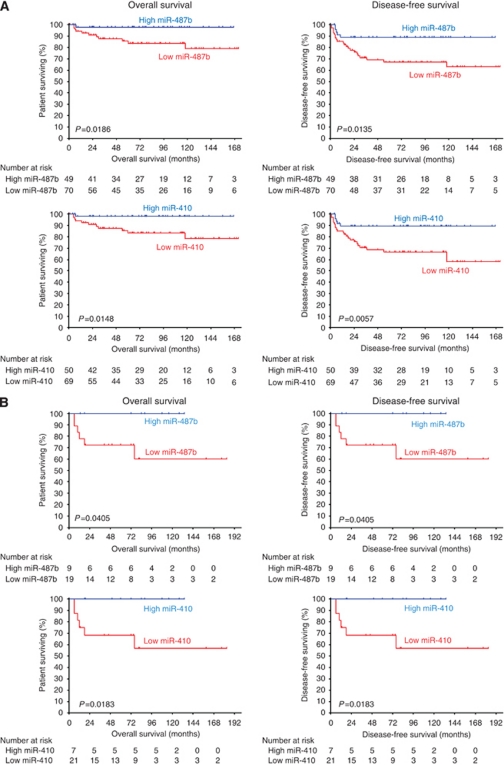
Kaplan–Meier curves for overall and disease-free survival of (**A**) non-*MYCN-*amplified localised neuroblastoma (*n*=119) and (**B**) non-*MYCN*-amplified stage 4S and 4 neuroblastoma of age <18 months (*n*=28) for miR-487b and miR-410 expression. The miRNA expression levels were converted into discrete variables into high and low classes, under or over the cutoffs. No relapse was observed in patients on (**B**).

Finally, we investigated the prognostic value of miR-487b and miR-410 expression levels in the subgroup of low-risk patients as defined by non-*MYCN*-amplified stage 1, 2 and 3 tumours. Kaplan–Meier survival curves revealed that the clinical outcome (overall and disease-free survival) of patients with high expression levels of either miR-487b or miR-410 was strongly correlated with better survival than patients with low expression levels (Figure 5A). Kaplan–Meier survival curves were also performed with the low-risk subgroup defined by non-*MYCN*-amplified tumours of stage 4 <18 months including stage 4S. Again, irrespective of the small number of samples (*n*=28), survival curves showed that patients with high expression levels of either miR-487b or miR-410 had a better prognosis in terms of overall and disease-free survival than those with low expression levels (Figure 5B).

## Discussion

A body of evidence demonstrates that some specific miRNAs can be considered as biomarkers for clinical applications especially for neuroblastoma (Stallings *et al*, 2010). In the search of a unique miRNA signature able to improve risk classification of these tumours, we identified a set encoded by the chromosome 14q32.31 miRNA cluster locus that was significantly downregulated in high-risk neuroblastoma. We report here that two of them, miR-410 and miR-487, which exhibited the highest fold change were able to discriminate high-risk from low-risk neuroblastoma and could be considered as putative prognostic factors. Moreover, expression of these two miRNAs was significantly associated with the overall survival and disease-free survival in the subgroup of neuroblastoma so far delineated as low-risk.

The wide spectrum of biological and clinical behaviours of neuroblastoma has resulted in basic risk stratification parameters (age, stage and *MYCN* amplification) that discriminate high-risk from low-risk patients (Cohn *et al*, 2009; Maris, 2010). However, limitations in this stratification system are generally recognised and addition of prognostic factors might be helpful to provide optimised and personalised neuroblastoma treatment. Most results dealing with miRNA in neuroblastoma concern the identification of miRNAs differentially expressed in *MYCN-*amplified *vs* non-*MYCN*-amplified tumours (Chen and Stallings, 2007; Bray *et al*, 2009). Dysregulation of some miRNAs encoded by the 14q32.31 locus has been already reported but the relationship between their abnormal expression and the clinical outcome of the disease has not been investigated (Bray *et al*, 2009). We performed microarray expression profiling on a preliminary cohort of 13 neuroblastoma tumours that allowed identification of 17 downregulated miRNAs that were significant discriminators of high-risk neuroblastoma. Notably, 15 were located at the 14q32.31 chromosomal locus. Three miRNAs were selected for further studies, miR-487b, miR-410, with the highest fold change, and miR-409-3p, with a medium fold change. The significant downregulation of these three miRNAs found in high-risk neuroblastoma was first confirmed by qRT-PCR on the preliminary cohort samples and then validated using an independent cohort of 214 tumours. To investigate the possible clinical use of these three miRNAs as potential biomarkers of aggressiveness, statistical analyses were performed on the whole retrospective cohort of the 227 neuroblastoma patients. Expression values were set up as binary variables coded as 0 for low expression and 1 for high. The results indicated that low expression levels of miR-487b, miR-410 and miR-409-3p were strongly and significantly associated with high-risk phenotype independent of *MYCN* amplification for miR-487b and miR-410, but not for miR409-3p. Moreover, expression levels of miR-487b were independent of clinical covariates (age, stage and *MYCN* amplification) associated with overall and disease-free survival in the multivariable model. Then, we surveyed the prognostic impact of miR-487b and miR-410 expression levels in each group of risk; miR-409-3p that was found not associated with non-*MYCN*-amplified high-risk tumours was not retained for further analyses. In high-risk neuroblastoma (*n*=80), we found that the expression of each miRNA was associated with overall survival, though with a trend of significance. By contrast, considering the two subgroups of low-risk neuroblastoma, that is, non-*MYCN*-amplified stage 1, 2 and 3 patients and non-*MYCN*-amplified stage 4 of infant <18 months, expression levels of both miR-487b and miR-410 displayed a highly significant association with overall and disease-free survival. Thus, our study clearly shows the clinical potential of miR-410 and miR-487b for the non-*MYCN*-amplified low-risk neuroblastoma. In combination with other markers of relapse, high levels of these two miRNAs could indicate the prescription of treatment des-escalation and low levels escalation. Whether the rest of top-ranked miRNAs of the 14q32.31 set, not considered in the present study, could help to discriminate unfavourable neuroblastoma remains to be investigated.

With regards to the high-risk group (*MYCN*-amplified and non-*MYCN-*amplified stage 4 ⩾18 months), the marginal significance of overall survival may be owing to the low number of survivors at 5 years post diagnosis. Noteworthy, most survivors showed a high expression level of miR-487b and miR-410. It remains thus to extend our investigation to a higher number of high-risk neuroblastoma.

From a functional point of view, the fact that 15/17 miRNAs (88%) significantly downregulated in high-risk neuroblastoma are encoded by the 14q32.31 locus suggests a tumour suppressive role for this chromosomal region; this is in agreement with reduced expression levels of these miRNAs measured in aggressive forms of several human cancers (Cobbs *et al*, 2008; Haller *et al*, 2010; Lavon *et al*, 2010). As the largest miRNA cluster identified so far in human (Glazov *et al*, 2008), the 14q32.31 locus encompasses >40 miRNAs likely encoded in a single pri-miRNA (Tierling *et al*, 2006). Noteworthy, this genetically imprinted region (Seitz *et al*, 2004) modulates neuronal responses with some miRNAs shown to have a crucial role in dendritic outgrowth in response to the environment (Khudayberdiev *et al*, 2009). In the same line of evidence, the mouse homologous region at chromosome 12 has been defined as a cancer susceptibility locus (Sevignani *et al*, 2007; Kircher *et al*, 2008). No loss of 14q copy in high-risk neuroblastoma samples suggests that the reduced expression of the encoded miRNAs is likely the result of a transcriptional or processing regulation, in agreement with reports stating the epigenetic transcriptional silencing of this genomic locus (Zhang *et al*, 2008; Haller *et al*, 2010; Lavon *et al*, 2010). Noteworthy, the proved target genes of 14q32.31 miRNAs are involved in numerous key biological functions such as transcription factors (E2F1, HNF-6 and NF-YB), cell cycle regulation (CDK1), proliferation (HER2), stress response (SOD1, 2), cytoskeleton signalling (PAK1) and cell interaction (E-Cadherin), of which dysregulation could lead to malignancy (Supplementary Table 6).

In conclusion, our results point out the involvement of 14q32.31 miRNAs in tumour progression of neuroblastoma. More importantly, our study suggests the potential of two miRNAs, miR-487b and miR-410, as clinically useful markers of both overall survival and disease-free survival in the two clinical entities delineated so far as low-risk, for example, non-*MYCN*-amplified stage 1, 2 and 3 and non-*MYCN*-amplified stage 4 <18 months. If our findings can be confirmed in other studies, miR-487b and miR-410 might be integrated as molecular markers in the stratification algorithm under investigation that would enable to optimise the treatment of those patients. To the best of our knowledge, no data are available on the potential of miRNAs to be used as predictors of relapse for low-risk patients and of survival for high-risk patients.

## Figures and Tables

**Table 1:** Differential miRNA expression between low- and high-risk neuroblastoma with adjusted *P*-value <0.05

**miRNAs**	**Chromosome location**	**Fold change low**/**high risk**	**Raw *P*-value**	**Adjusted *P*-value**
miR-487b	14q32.31	5.75	2.36 × 10^−6^	9.38 × 10^−4^
miR-149	2q37.3	4.69	3.59 × 10^−4^	1.30 × 10^−2^
miR-410	14q32.31	3.97	1.33 × 10^−4^	7.53 × 10^−3^
miR-331-3p	12q22	3.79	6.94 × 10^−5^	5.51 × 10^−3^
miR-323-3p	14q32.31	3.61	2.38 × 10^−7^	1.89 × 10^−4^
miR-758	14q32.31	3.20	1.94 × 10^−5^	2.92 × 10^−3^
miR-299-5p	14q32.31	3.08	1.15 × 10^−4^	7.03 × 10^−3^
miR-654-3p	14q32.31	3.00	1.32 × 10^−5^	2.63 × 10^−3^
miR-495	14q32.31	2.98	8.79 × 10^−5^	5.87 × 10^−3^
miR-409-3p	14q32.31	2.69	4.97 × 10^−4^	1.58 × 10^−2^
miR-377	14q32.31	2.37	7.92 × 10^−4^	2.19 × 10^−2^
miR-485-3p	14q32.31	2.34	4.14 × 10^−4^	1.43 × 10^−2^
miR-543	14q32.31	2.26	8.87 × 10^−5^	5.87 × 10^−3^
miR-409-5p	14q32.31	2.25	6.83 × 10^−5^	5.51 × 10^−3^
miR-329	14q32.31	2.25	5.10 × 10^−5^	5.07 × 10^−3^
miR-154	14q32.31	2.11	2.67 × 10^−4^	1.12 × 10^−2^
miR-154^*^	14q32.31	2.06	5.88 × 10^−4^	1.73 × 10^−2^

Abbreviation: miRNA=microRNA.

**Table 2:** Characteristics of patients and tissues

	**Preliminary cohort (%)**	**Validation set (%)**	**Total (%)**
*Number of tissues*	13	214	227^a^
Stage			
1	1 (8)	33 (15)	34 (15)
2	2 (15)	45 (21)	47 (21)
3	2 (15)	46 (21)	48 (21)
4	7 (54)	74 (36)	81 (36)
4S	1 (8)	16 (7)	17 (7)
			
Age at diagnosis			
<18 months	8 (62)	101 (47)	109 (48)
⩾18 months	5 (38)	113 (53)	118 (52)
			
MYCN status			
Amplified	8 (62)	23 (11)	31 (14)
Non-amplified	5 (38)	191 (89)	196 (86)
			
*Number of patients*			226^a^
Present status			
Alive	6 (46)	152 (71)	158 (70)
Death of disease	7 (54)	62 (29)	68 (30)
			
Disease-free status			
Disease-free	4 (31)	132 (62)	136 (60)
Disease	9 (69)	82 (38)	90 (40)

a Two tumour specimens were obtained from the same patient at different time of resection, one at diagnosis (validation set), the second at relapse (preliminary cohort).

**Table 3:** Univariate and multivariable Cox regression analysis of overall and disease-free survival in all neuroblastoma patients (*n*=226)

	**Univariate analysis**		**Multivariable analysis with miR-487b**		**Multivariable analysis with miR-410**	
	**Hazard ratio (95% CI)**	***P*-value**	**Hazard ratio (95% CI)**	***P*-value**	**Hazard ratio (95% CI)**	***P*-value**
*Overall survival*						
Age at diagnosis						
>18 months	1.0 (reference)	<0.0001	1.0 (reference)	0.0441	1.0 (reference)	0.0258
<18 months	0.247 (0.139–0.440)		0.521 (0.276–0.983)		0.485 (0.257–0.917)	
						
Stage						
4	1.0 (reference)	<0.0001	1.0 (reference)	0.0001	1.0 (reference)	<0.0001
1, 2, 3, 4S	0.160 (0.093–0.275)		0.294 (0.158–0.547)		0.279 (0.148–0.526)	
						
MYCN status						
Amplified	1.0 (reference)	<0.0001	1.0 (reference)	0.0704	1.0 (reference)	0.0376
Non-amplified	0.288 (0.171–0.487)		0.601 (0.346–1.043)		0.554 (0.318–0.967)	
						
MiR-487b expression						
High	1.0 (reference)	0.0001	1.0 (reference)	0.0183		
Low	5.129 (2.218–11.861)		2.833 (1.192–6.731)			
						
MiR-410 expression						
High	1.0 (reference)	0.0034			1.0 (reference)	0.2906
Low	2.623 (1.375–5.003)				1.448 (0.729–2.875)	
						
*Disease-free survival*						
Age at diagnosis						
>18 months	1.0 (reference)	0.0002	1.0 (reference)	0.1678	1.0 (reference)	0.0990
<18 months	0.429 (0.274–0.672)		0.703 (0.427–1.160)		0.656 (0.397–1.083)	
						
Stage						
4	1.0 (reference)	<0.0001	1.0 (reference)	0.0014	1.0 (reference)	0.0016
1, 2, 3, 4S	0.307 (0.200–0.470)		0.445 (0.270–0.731)		0.443 (0.268–0.735)	
						
MYCN status						
Amplified	1.0 (reference)	0.0016	1.0 (reference)	0.3112	1.0 (reference)	0.2479
Non-amplified	0.449 (0.273–0.738)		0.762 (0.450–1.290)		0.733 (0.432–1.242)	
						
MiR-487b expression						
High	1.0 (reference)	0.0002	1.0 (reference)	0.0144		
Low	3.036 (1.686–5.466)		2.145 (1.164–3.952)			
						
MiR-410 expression						
High	1.0 (reference)	0.0026			1.0 (reference)	0.0912
Low	2.253 (1.328–3.820)				1.614 (0.926–2.813)	

Abbreviation: CI=confidence interval.

**Table 4:** Sensitivity and specificity of the miR-487b marker in all neuroblastoma patients (*n*=226) with 156 ‘low expression’ and 70 ‘high expression’

**Outcome**	**Within**	**Sensitivity^a^**	**Specificity^a^**
DoD	2 years	0.87	0.34
R or DoD		0.84	0.36
DoD	5 years	0.89	0.38
R or DoD		0.85	0.4
DoD	8 years	0.9	0.41
R or DoD		0.88	0.45

Abbreviations: DoD=death of disease; R=relapse.

a Sensitivity and specificity are estimated through the Kaplan–Meier estimator.
