# Supplementary material for: Expression of miR-487b and miR-410 encoded by 14q32.31 locus is a prognostic marker in neuroblastoma
Source: Br J Cancer. 2011 Oct 4;105(9):1352–61. doi: 10.1038/bjc.2011.388 (PMC3241557; doi:10.1038/bjc.2011.388)
Supplement: Supplementary Figure 1 [file bjc2011388x1.ppt]

## Slide 1
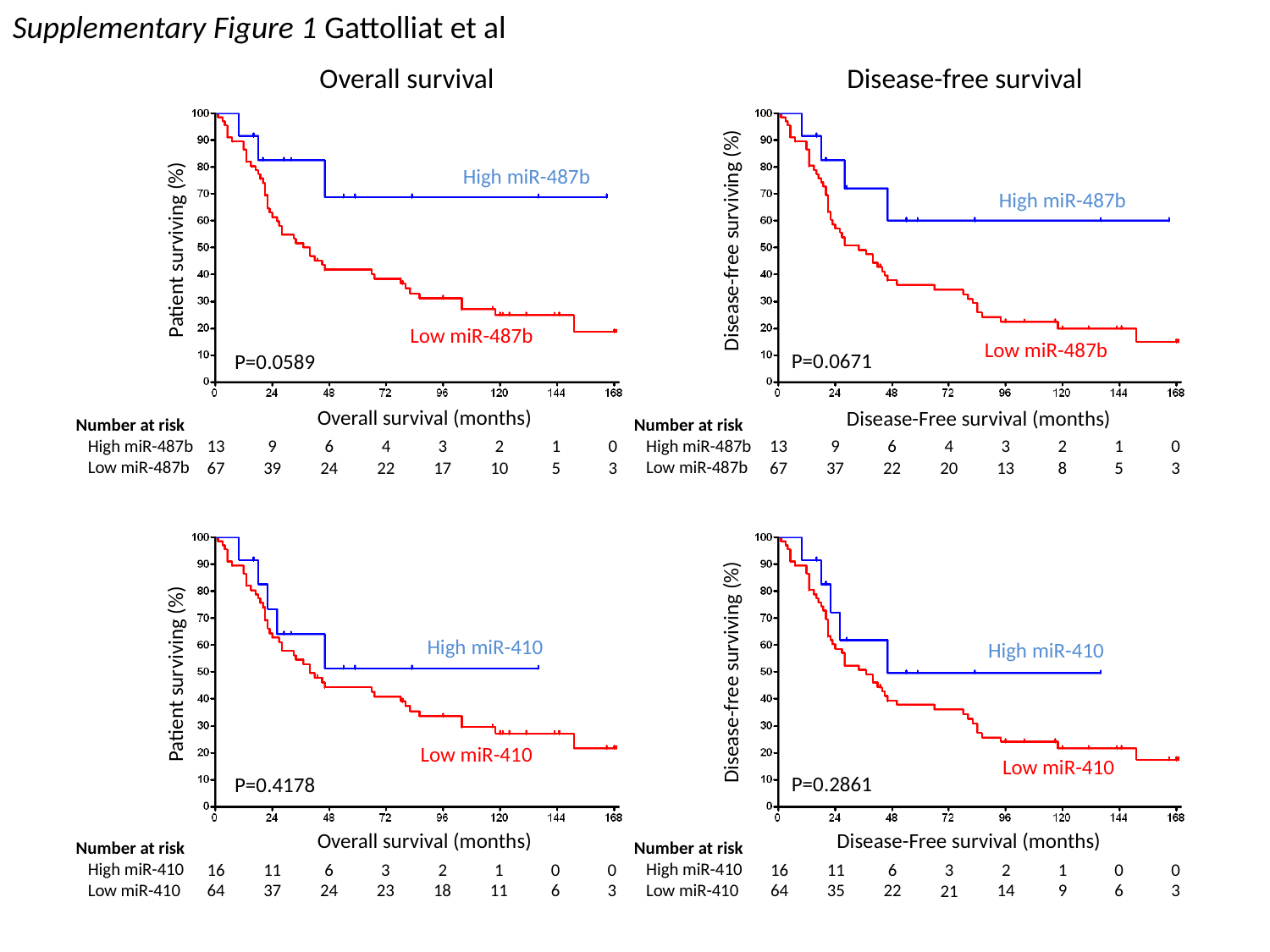

Supplementary Figure 1 Gattolliat et al
Overall survival
Disease-free survival
High miR-487b
High miR-487b
Disease-free surviving (%)
Patient surviving (%)
Low miR-487b
Low miR-487b
P=0.0671
P=0.0589
Overall survival (months)
Disease-Free survival (months)
Number at risk
 High miR-487b
 Low miR-487b
Number at risk
 High miR-487b
 Low miR-487b
13
67
9
39
6
24
4
22
3
17
2
10
1
5
0
3
13
67
9
37
6
22
4
20
3
13
2
8
1
5
0
3
High miR-410
High miR-410
Disease-free surviving (%)
Patient surviving (%)
Low miR-410
Low miR-410
P=0.2861
P=0.4178
Overall survival (months)
Disease-Free survival (months)
Number at risk
 High miR-410
 Low miR-410
Number at risk
 High miR-410
 Low miR-410
16
64
11
37
6
24
3
23
2
18
1
11
0
6
0
3
16
64
11
35
6
22
2
14
1
9
0
6
0
3
3
21
