# Supplementary material for: Expression of miR-487b and miR-410 encoded by 14q32.31 locus is a prognostic marker in neuroblastoma
Source: Br J Cancer. 2011 Oct 4;105(9):1352–61. doi: 10.1038/bjc.2011.388 (PMC3241557; doi:10.1038/bjc.2011.388)
Supplement: Supplementary Table 1 [file bjc2011388x2.doc]

**Supplementary Table 1** Genomic characteristics of tumors used in the preliminary cohort

| Risk at diagnosis | High | | | | | | | | Low | | | | |
| --- | --- | --- | --- | --- | --- | --- | --- | --- | --- | --- | --- | --- | --- |
| Patient Code | 104 | 301 | 209 | 181 | 358 | 305 | 600 | 2232 | 424 | 343 | 459 | 118 | 2192 |
| CGH Curies’s stratification^a^ | C | B | C | C | C | C | C | C | A | A | A | NO | D |
| 1p loss^b^ | yes | no | yes | yes | yes | no | yes | no | no | no | no | no | no |
| 1q gain^b^ | no | no | no | no | no | no | no | no | no | no | no | no | no |
| 3p loss^b^ | no | no | no | no | no | no | no | no | no | no | no | no | no |
| 7q gain^b^ | yes | yes | yes | no | no | no | no | no | no | no | no | no | no |
| 11q loss^b^ | yes | no | no | no | no | no | no | no | no | no | no | no | no |
| 17q gain^b^ | yes | no | yes | no | no | no | yes | no | no | no | no | no | no |
| 14q32.31 loss^b^ | 0 | 0 | 0 | 0 | 0 | 0 | -0.36 | 0 | 0 | 0 | -0.16 | -0.19 | -0.19 |

^a^using CGH-array; A, B, C and D types according to neuroblastoma stratification with **A**: numerical aberrations, no segmental aberrations; **B**: segmental aberrations, no numerical aberrations; **C**: *MYCN* amplification, no numerical aberrations; **D**: segmental and numerical aberrations; ^b^obtained using CGH-array (Janoueix-Lerosey et al., 2009); +: yes .

**Array-based CGH for Genomic DNA Analysis Protocol Version 4.0**

*Sample preparation and hybridization.* Before labelling and hybridization, genomic DNA (0.2-0.5 µg) was fragmented by a double enzymatic digestion with *Alu*I and *Rsa*I and checked with LabOnChip (*2100 Bioanalyzer System,* Agilent Technologies). Control DNA from Promega (Human Genomic DNA Female G1521) and tumor DNAs were labelled by random priming with CY5-dCTP and CY3-dCTP using Labelling Kit PLUS (*Agilent p/n 5188-5309*). The DNA hybridization was carried out for 17 hours at 65°C in a rotating oven (Robbins Scientific, Mountain View, CA) at 20 rpm using a clean gasket slide (*Agilent p/n G2534-60013*) and then covered with the *Agilent* 244K microarray containing an Agilent Oligo aCGH/ChIP-Chip Hybridization Kit (*Agilent p/n 5188-5220*). The chips were scanned on an Agilent Technologies G2505C.

*Normalization.* Signal acquisition and normalization from the scanned image was performed using the Feature Extraction software version 10.1.1.1 from *Agilent* Technologies, with protocol CGH-v4_10_Apr08, and the array design version 014693_D_ 20080627, both from Agilent Technologies. Default settings were used within the software. Normalized data were re-centralized using a custom script, then analyzed using CGH Analytics 3.4.40 software under the following parameters: ADM-2 used as the segmentation method, with a threshold of 10 [ref_ADM2]. Aberration calling was performed using an aberration-level filter of 5 consecutive probes for an absolute log2 (ratio) > 0.15. The UCSC human genome build version hg18 of March 2006 was used for all the genomic coordinates [http://genome.ucsc.edu].

**Reference**

Janoueix-Lerosey I, Schleiermacher G, Michels E, et al. Overall genomic pattern is a predictor of outcome in neuroblastoma. *J Clin Oncol* 2009; 27: 1026-33.
