# Supplementary material for: Expression of miR-487b and miR-410 encoded by 14q32.31 locus is a prognostic marker in neuroblastoma
Source: Br J Cancer. 2011 Oct 4;105(9):1352–61. doi: 10.1038/bjc.2011.388 (PMC3241557; doi:10.1038/bjc.2011.388)
Supplement: Supplementary Table 6 [file bjc2011388x7.doc]

**Supplementary Table 6** Proved target genes and their known involvement in neuroblastoma

| **MicroRNA** | **Proved targets** | **Neuroblastoma involvement of the targets** |
| --- | --- | --- |
| miR-410 | CDK1 |  |
| miR-331-3p | HER2  E2F1 | - Upregulated in neuroblastoma^1^  - Upregulated in stage 4 non-*MYCN*-amplified tumors vs stage 1 tumor^2^  - Downregulated in tumours with a favourable prognosis vs unfavourable^3^ |
| miR-299-5p | OPN | - Upregulated in neuroblastoma^1^  - High in unfavorable neuroblastoma^4^ |
| miR-654-3p | CDKN1A | - Upregulated in neuroblastoma^1^  - High in unfavorable neuroblastoma^4^ |
| miR-495 | E-cadherin  OC-2  REDD1  HNF-6 | - Negatively correlated with overall survival^5^ |
| miR-409-3p | FGB |  |
| miR-377 | SOD1  SOD2  PAK1 | - Higher expressed in unfavorable neuroblastoma^6^ |
| miR-485-3p | NF-YB |  |

**Reference List**

1 - Roberts SS, Mori M, Pattee P, et al. GABAergic system gene expression predicts clinical outcome in patients with neuroblastoma. J Clin Oncol 2004; 22: 4127-34.

2 - Krasnoselsky AL, Whiteford CC, Wei JS*, et al*. Altered expression of cell cycle genes distinguishes aggressive neuroblastoma. Oncogene 2005; 24: 1533-41.

3 - Warnat P, Oberthuer A, Fischer M*, et al*. Cross-study analysis of gene expression data for intermediate neuroblastoma identifies two biological subtypes. BMC Cancer 2007; 7: 89.

4 - Abel F, Sjoberg RM, Nilsson S, Kogner P, Martinsson T. Imbalance of the mitochondrial pro- and anti-apoptotic mediators in neuroblastoma tumours with unfavourable biology. Eur J Cancer 2005; 41: 635-46.

5 - Oberthuer A, Kaderali L, Kahlert Y*, et al*. Subclassification and individual survival time prediction from gene expression data of neuroblastoma patients by using CASPAR. Clin Cancer Res 2008; 14: 6590-601.

6 - Hiyama E, Hiyama K, Yamaoka H*, et al*. Expression profiling of favorable and unfavorable neuroblastomas. Pediatr Surg Int 2004; 20: 33-8.
