# Supplementary material for: Expression of miR-487b and miR-410 encoded by 14q32.31 locus is a prognostic marker in neuroblastoma
Source: Br J Cancer. 2011 Oct 4;105(9):1352–61. doi: 10.1038/bjc.2011.388 (PMC3241557; doi:10.1038/bjc.2011.388)
Supplement: Supplementary Information [file bjc2011388x8.doc]

**Supplementary online material**

***Supplementary Figure 1*** Kaplan-Meier curves for overall survival of high-risk neuroblastoma (n=80) for miR-487b and miR-410 expression. miRNAs expression levels were converted into discrete variables by discriminating the samples into two classes (high and low), under or over the cut-offs.

***Supplementary Table 1*** Genomic characteristics of tumours used in the preliminary cohort

***Supplementary Table 2*** Differential miRNA expression between high-risk (n=8) and low-risk (n=5) neuroblastoma with raw-p-value <0.05.

***Supplementary Table 3*** Differential 14q32.31 miRNA expression of the preliminary cohort.

***Supplementary Table 4*** Main characteristics of the preliminary and validation sets and the expression levels of the three selected miRNAs using qRT-PCR

***Supplementary Table 5*** Relationship between various clinical characteristics and 14q32.31 miRNA expression levels using two-tailed Fisher's exact test

***Supplementary Table 6*** Proved target genes and their known involvement in neuroblastoma
